# Transapical transcatheter device closure of relapse left ventricular diverticulum: a novel minimally invasive technique for reoperation

**DOI:** 10.1186/s13019-016-0546-4

**Published:** 2016-11-16

**Authors:** Weizhi Zhang, Lei Gao, Yifeng Yang, Tianli Zhao

**Affiliations:** Department of Cardiovascular Surgery, The Second Xiangya Hospital of Central South University, 139 Middle Renmin Road, Changsha, Hunan 410011 China

**Keywords:** Cardiac reoperation, Minimally invasive technique, Echocardiography

## Abstract

We describe a novel minimally invasive technique, transapical transcatheter device closure of relapse left ventricular diverticulum (LVD) under transesophageal echocardiography (TEE) guidance. The patient previously underwent LVD primary repair and mitral valvular replacement. The anatomical characteristics of the relapse LVD and its adjacent structures are detailedly delineated by echocardiography. The relapse LVD is then managed by transapical transcatheter device closure under TEE guidance. This novel minimally invasive technique is not only limited in relapse LVD, but also could be extensively applied in other cardiac reoperation, such as perivalvular leak closure after valvular replacement.

Dear Sir,

A 12-year-old Chinese boy presents to us with exercise-induced dyspnea for 3 weeks. He underwent an open-heart surgery one year before. Diagnosis of isolated congenital left ventricular diverticulum (LVD) with mitral valve infective endocarditis was made intraoperatively, and the diverticulum was successfully repaired by direct suturing the orifice, and mitral valve was replaced with a 23 mm inverted supra-annular mechanical valve. Transthoracic echocardiography (TTE) was performed at discharge, one month and six months follow-ups, and all of which confirmed obliteration of the diverticulum with good function of mechanical valve and left ventricle (LV). On this admission, his heart rate is 110 bpm, the cardiac dullness is extended to the left and downward, and there is no heart murmur. Chest x-ray film shows cardiomegaly (Fig. 1a). TTE shows markedly impaired LV function (EF = 38%, FS = 19%), and a saccular structure (38 × 39 mm) localized between the left atrium and the aortic root, communicating with the LV through a 13 mm neck (Fig. 1b), which are similar to the findings before last surgery, suggestive of the relapse of the LVD.

**Fig. 1:**
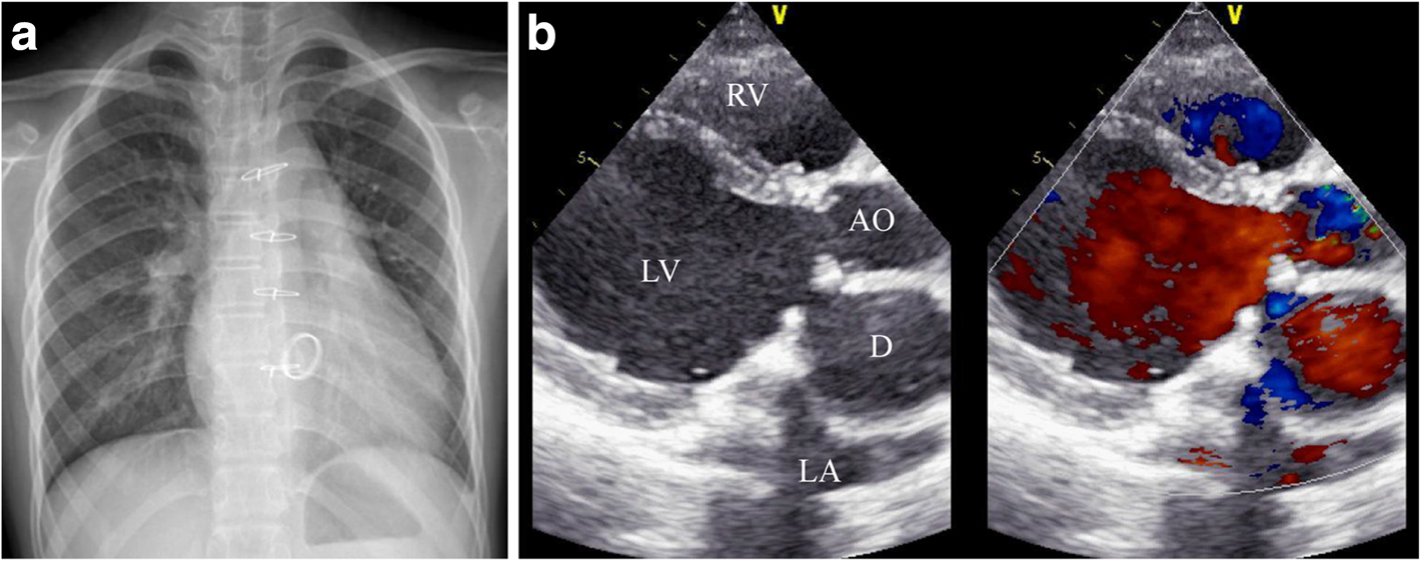
Multimodality imaging of the relapse left ventricular diverticulum (D). **a**. Chest x-ray film shows cardiomegaly. **b**. Transthoracic echocardiography shows a saccular structure (D) localized between the left atrium (LA) and the aortic root (AO), communicating with the left ventricle (LV) through a 13 mm wide neck

To rescue the LV function resulted from the relapse LVD, we develop a novel minimally invasive technique, transapical transcatheter LVD device closure. Transesophageal echocardiography (TEE) is applied to perform a comprehensive study after general anesthesia and intubation, looking at all aspects of the LVD anatomy (location, size, neck and its adjacent structure) (Fig. 2a). The LVD neck is sized with the maximum diameter, which is 13 mm. And a 16 mm atrial septal defect (ASD) occluder (Shanghai Shape Memory Alloy Co. Ltd., Shanghai, China) is chosen for LVD neck closure. The LV apex is identified by palpation and TTE. The pleural space overlying the apex is entered via a 3 cm anterolateral thoracotomy. The pericardium over the apex is identified and dissected. The thin portion of the apex is identified by finger palpation and confirmed by simultaneous TEE, and then a purse-string suture with pledget is placed. After puncture of the left ventricular apex, the guidewire is passed through the LVD neck into the diverticulum under TEE constant guidance. The delivery sheath is advanced into the diverticulum, just across the LVD neck. The guidewire is removed and the delivery sheath is filled with blood to ensure de-airing (Fig. 2b). The loading sheath is then connected, and the ASD occluder is advanced. The device is released step-by-step under TEE guidance, with left disk deployed in the diverticulum (Fig. 2c), while the right disk in the LV (Fig. 2d). After confirmation of an adequate occlusion of the LVD neck, together with the documentation of undisturbed adjacent aortic and mitral valve function, the loading cable is released and the loading system is retrieved. Hemostasis is secured with the previously placed pledgeted suture. The chest is closed in a routine fashion. After an uneventful recovery the patient is discharged home on the 5th postoperative day, when the TTE confirms an adequate occlusion of the LVD, with improved LV function (EF = 52%, FS = 27%). No hydropericardium or hydrothorax was seen by TTE. Eight months of patient follow-ups do not show any discomfort, and last TTE indicates EF = 67%, FS = 38%.

**Fig. 2:**
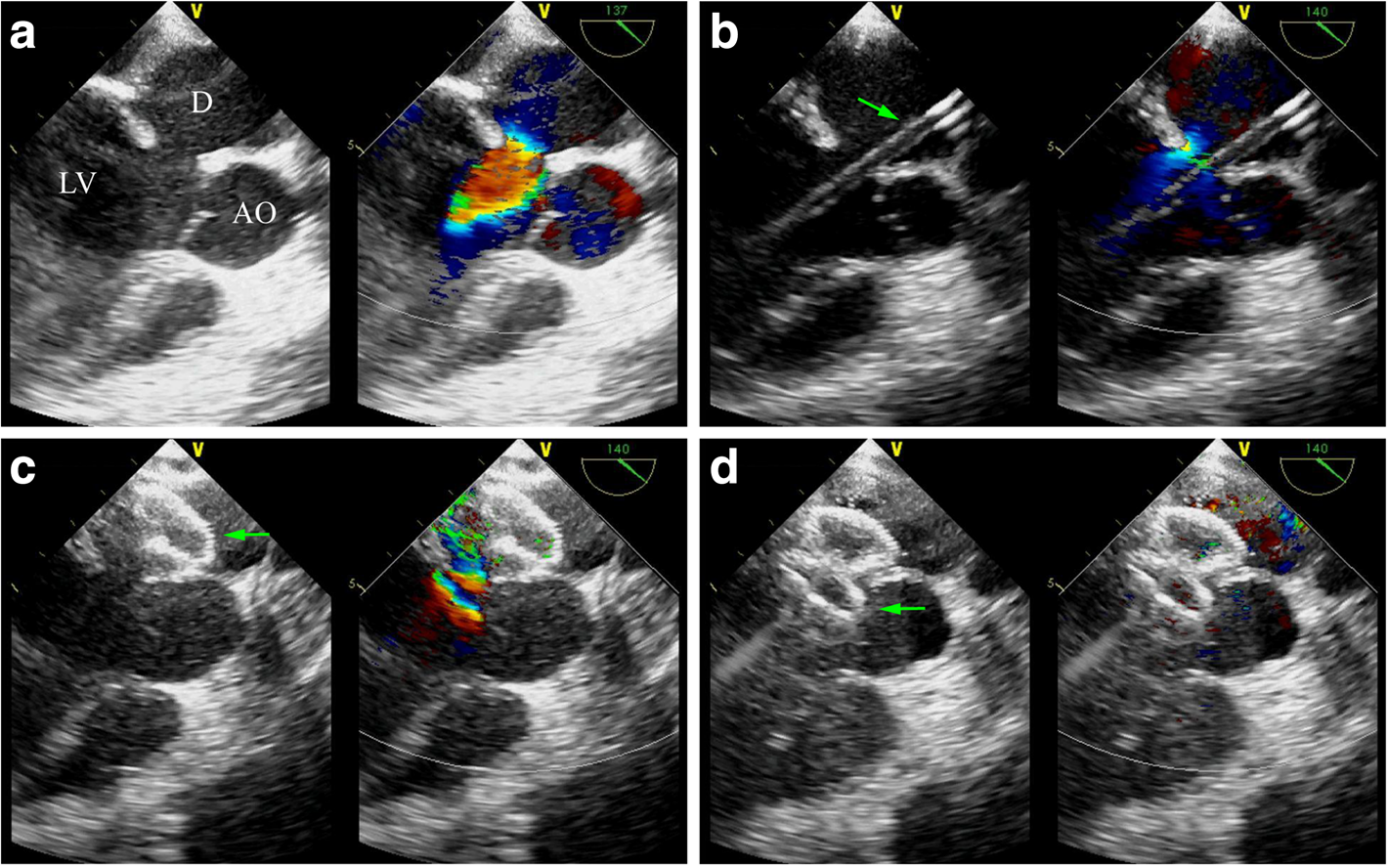
Surgical approach of transapical transcatheter device closure of relapse *left* ventricular diverticulum under transesophageal echocardiography (TEE) guidance. LV, *left* ventricle; AO, aorta; D, *left* ventricular diverticulum

The mechanism of the in situ relapse of the LVD in our case is unknown, though the fracture of the suture should be the most supposed reason [1]. We prefer a new minimally invasive technique, transapical transcatheter LVD device closure, rather than conventional re-open heart surgery in this case for several reasons. First, the deep-seated localization of the LVD, makes the dissection and surgical resection of the diverticulum technically difficult during a reoperation. Also, the exposure and suturing of the orifice can be problematic if repair the LVD inside LV, because of the mechanical mitral valve implanted in the first operation. Second, the minimally invasive technique permits the avoidance of a re-sternotomy and re-cardiotomy. Instead, only a direct puncture in the left ventricular apex is performed. Third, this technique avoids cardiopulmonary bypass. In the meanwhile, we always keep in mind the potential complications, such as rupture of diverticulum, valve injury, device drop-off, embolism, and arrhythmia. The successful application of the transapical transcatheter device closure in the relapse LVD case demonstrates the usefulness and feasibility of this new technique. Moreover, this technique is not only limited in relapse LVD, but also could be extensively applied in other cardiac reoperation, such as perivalvular leak closure after valvular replacement.

## Abbreviations

ASD: Atrial septal defect
LV: Left ventricle
LVD: Left ventricular diverticulum
TEE: Transesophageal echocardiography
TTE: Transthoracic echocardiography
